# Myocardial tissue characterisation using echocardiographic deformation imaging

**DOI:** 10.1186/s12947-019-0176-9

**Published:** 2019-11-15

**Authors:** Mohammed A. Moharram, Regis R. Lamberts, Gillian Whalley, Michael J. A. Williams, Sean Coffey

**Affiliations:** 1 Department of Medicine – HeartOtago, Dunedin School of Medicine, University of Otago, Box 56, Dunedin, PO 9054 New Zealand; 2 Department of Physiology – HeartOtago, School of Biomedical Sciences, University of Otago, Dunedin, New Zealand

**Keywords:** Echocardiography, Strain, Speckle tracking, Fibrosis, Myocardial histology

## Abstract

Myocardial pathology results in significant morbidity and mortality, whether due to primary cardiomyopathic processes or secondary to other conditions such as ischemic heart disease. Cardiac imaging techniques characterise the underlying tissue directly, by assessing a signal from the tissue itself, or indirectly, by inferring tissue characteristics from global or regional function. Cardiac magnetic resonance imaging is currently the most investigated imaging modality for tissue characterisation, but, due to its accessibility, advanced echocardiography represents an attractive alternative. Speckle tracking echocardiography (STE) is a reproducible technique used to assess myocardial deformation at both segmental and global levels. Since distinct myocardial pathologies affect deformation differently, information about the underlying tissue can be inferred by STE. In this review, the current available studies correlating STE deformation parameters with underlying tissue characteristics in humans are examined, with separate emphasis on global and segmental analysis. The current knowledge is placed in the context of integrated backscatter and the future of echocardiographic based tissue characterisation is discussed. The use of these imaging techniques to more precisely phenotype myocardial pathology more precisely will allow the design of translational cardiac research studies and, potentially, tailored management strategies.

## Introduction

**Fig. 1:**
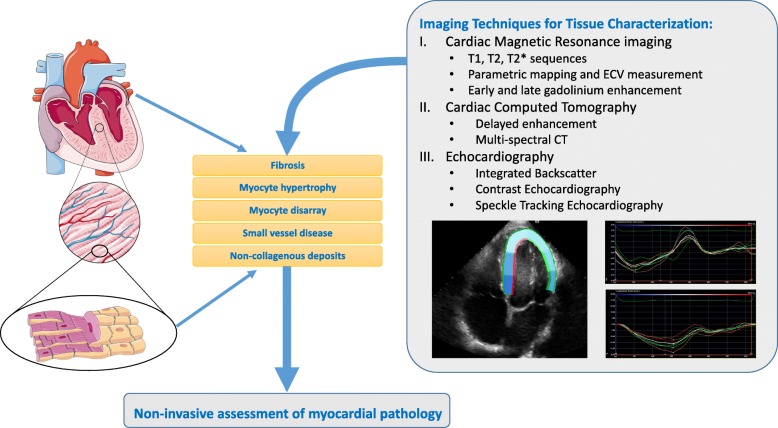
Overview of imaging techniques for tissue characterisation. Accurate characterisation of important myocardial features, such as fibrosis or myocyte hypertrophy, would allow non-invasive diagnosis of myocardial pathology, potentially allowing the development of personalized therapies. (Image contains material licensed under CC-BY 3.0 from https://smart.servier.com/). Abbreviations: CT, computed tomography; ECV, extracellular volume

The primary aim of non-invasive cardiac imaging is to provide information on the diagnosis and severity of underlying cardiac conditions. Many myocardial pathologies are distributed non-homogeneously and thus diagnosis may be unreliable using global assessments (such as ejection fraction or global longitudinal strain). More advanced characterisation of the underlying tissue, such as with cardiac magnetic resonance (CMR) imaging may allow more accurate diagnosis (Fig. 1). With notable exceptions (such as bone scintigraphy in amyloidosis [1]), cardiac magnetic resonance (CMR) imaging currently occupies this niche in cardiac imaging, with tissue characterisation performed using late gadolinium enhancement imaging and methods such as parametric and non-parametric T1, T2 and T2* imaging [2]. However, echocardiography remains the most common cardiac imaging procedure performed in clinical practice, due to its portability, low cost, and patient acceptance. Although often not recognized as such [3], tissue characterisation is already performed using echocardiography – a thinned and akinetic myocardium signifies transmural myocardial fibrosis, for instance, and there is significant research into ultrasonic backscatter. However, more advanced echocardiography methods, providing additional information on the structure and function of the underlying tissue, could be used in both translational and clinical research, and ultimately translated to clinical practice.

**Fig. 2:**
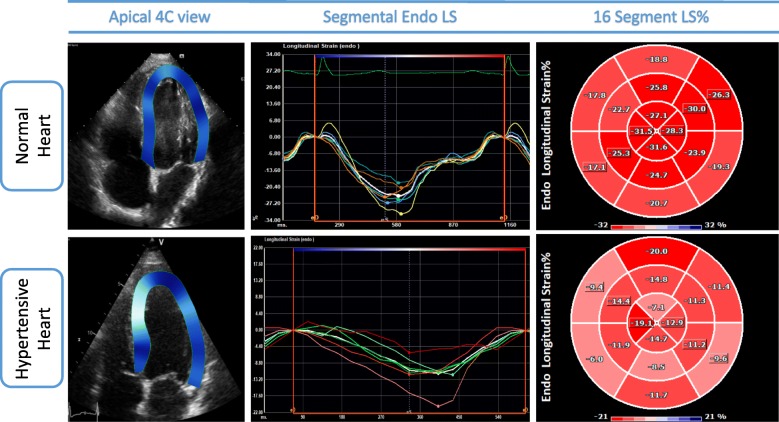
Segmental Speckle Tracking longitudinal strain analysis. Segmental analysis of longitudinal strain in a normal heart (top row) compared to a patient with advanced hypertensive heart disease with reduced longitudinal strain (lower row). The longitudinal strain curves show deformation of individual segments which is reflected in the 16-segment Bull’s eye. In this case, segmental strain is relatively globally impaired due to the homogeneous myocardial distribution of the condition. Abbreviations: Apical 4C, apical four chamber view; Endo, endocardial, LS, longitudinal strain

One such method is speckle tracking-echocardiography (STE). The tracking of grayscale speckles is independent from insonation angle and partially independent from translational movement of the heart, allowing myocardial deformation analysis without being restrained by the limitations of earlier technologies that were dependent on tissue velocity [4]. The most commonly used measurements are those of strain (the change in length compared to initial length), strain rate (SR, strain divided by time), and rotation (or twist). Strain measures can be reported for localized areas of interest (segmentally, Fig. 2), or measured over the whole chamber assessed (global). A recent excellent review covers deformation measures in more detail [4].

Deformation of a particular myocardial segment is dependent on cardiomyocyte function, myofibre architecture, tissue elasticity and fibrosis, as well as being affected by intracavity pressure and the function of neighboring segments [5]. In this narrative review, we examine the utility of STE deformation imaging for tissue characterisation, focusing on studies comparing imaging with the gold standard of ex-vivo tissue analysis, rather than on comparison with other imaging modalities.

## STE deformation for tissue characterisation

While other modalities such as CMR assess tissue characteristics through changes in the acquired myocardial tissue images [6], STE deformation parameters assess the impact of underlying pathology on tissue function [7]. Histological and pathophysiological changes affecting the extracellular matrix, cardiomyocyte contractile and/or regulatory proteins influence myocardial mechanics. Previously, the role of the extracellular matrix (ECM) in myocardial contraction and stiffness has been highlighted [8, 9], and changes in the ECM increase myocardial stiffness even after preventing myocyte hypertrophy in preclinical models [10]. On the cellular level, the changes affecting contractile and regulatory proteins have been shown to impact cardiomyocyte mechanics [11, 12]. These changes in cardiomyocyte mechanics are reflected in the deformation parameters assessed by STE, which can detect and quantify the amount as well as the rate of deformation of myocardial segments and is linked to a particular pattern of change in the myocardial tissue [13–15]. The concept of myocardial strain was initially introduced to help understand elastic stiffness of myocardial tissue [7]; strain rate represents the shortening velocity of myocardial fibre, which can be expressed as a force-velocity-length relation [16]. Strain and SR can be used as a contractility measure; however, STE can detect a specific pattern of change comprising various deformation parameters, which can be used to characterise underlying tissue histopathological changes.

### Global STE deformation for tissue characterisation

STE deformation can be assessed on both global and segmental levels [17]. Global deformation measures have been compared to histological examination in a limited number of studies (Table 1) and are based on the assumption that the pathological process affects the myocardium of the assessed chamber in a global and even manner. This is not true for patients with hypertrophic cardiomyopathy (HCM), and it is therefore no surprise that global LV deformation parameters showed no significant change in studies (in patients with preserved LV ejection fraction (EF)) investigating the incidence of ventricular arrhythmias [18], myocardial fibrosis [13, 19] and histopathological changes pertinent to HCM [13]. However, in the same studies, septal deformation parameters showed significant correlations with the corresponding pathologies [13, 18, 19].

**Table 1:** Summary of studies correlating global deformation parameters with tissue characteristics

Study	Patients and Pathology	Assessed global measures	Assessed Segmental measures	Tissue correlates	Correlations between global measures and tissue correlates	Correlations between segmental measures and tissue correlates
Almaas et al. 2013 (18).	63 patients. 24 patients had septal myectomy samples analysed. HCM with/without ventricular arrhythmia.	GLS (n = 63); HCM without ventricular arrhythmias (mean(SD)) -14.7 (3.4), with ventricular arrhythmias −12.2 (3.7).	SSL (*n* = 63); HCM without ventricular arrhythmia (mean(SD)) -13.6 (5.6), with ventricular arrhythmia - 9.0 (4.0).	Fibrosis (perivascular, interstitial, subendocardial, replacement).	–	SSL (*n* = 24); total fibrosis (R^2^ 0.31, *P* < 0.05), interstitial fibrosis (R^2^ 0.36, *P <* 0.05), replacement fibrosis (R^2^ 0.03, NS).
Almaas et al. 2014 (19).	HCM (*n* = 32).	(Total fibrosis> = 15%) GLS (OR 1.27, NS), GCS (OR 1.08, NS).	(Total fibrosis> = 15%) Septal LS (OR 1.38, *P* < 0.05), (Multivariate OR 1.79, *P* < 0.05). Septal CS (OR 1.06, NS).	Fibrosis (Total, interstitial, replacement).	–	SSL with fibrosis: total (r = 0.50, *P* < 0.05), interstitial (r = 0.40 *P* < 0.05), replacement fibrosis (r = 0.28, NS).
Kobayashi et al. 2013 (13).	HCM (*n* = 171).	3 subgroups: HCM without hypertension, HCM with hypertension, and hypertensive heart disease without HCM. GLSR (mean(SD)) (−1.05 (0.3), − 1.01 (0.3), and − 1.14 (0.3), NS respectively) and SRe (1.03 (0.4), 0.96 (0.4), and 1.09 (0.3), NS, respectively).	3 subgroups: HCM without hypertension, HCM with hypertension, and hypertensive heart disease without HCM. Basal SSRs (mean(SD)) (− 0.87 (0.5), − 0.95 (0.5), and − 0.98 (0.4), NS, respectively) and SSRe (0.76 (0.5), 0.86 (0.5), and 0.82 (0.5), NS, respectively).	Myocyte hypertrophy, myocyte disarray, SICAD, interstitial fibrosis.	–	SSRe, myocyte disarray −0.19, *P* < 0.05. SSRs, myocyte hypertrophy 0.21, *P* < 0.05, myocyte disarray 0.23 *P* < 0.05.
Witjas-Paalberends, et al. 2014 (11).	HCM (*n* = 46).	GLS (mean(SD)) was reduced in both HCM_MUT_ (− 16.0 (3.2)%) and HCM_SMN_ (− 15.1 (3.1)%) compared with controls (− 21.0 ( 3.2)%, *P* < 0.001 and *P* < 0.05 respectively).	SSL (mean(SD)) (%) HCM_MUT_ (−7.0 (4.3) *P* < 0.001, SSRs(1/s) -0.64 (0.58), *P* < 0.05, SSRe (1/s) 0.47 (0.33) *P* < 0.001 versus controls).	Cardiomyocyte maximal developed tension.	–	Basal SSL: maximal tension (Spearman’s ρ 0.46, *P* < 0.05).
Park et al. 2019 (23).	Severe AS (*n* = 71)	GLS (mean(SD)) (fibrosis, mild − 16.30 (2.97), moderate − 14.76 (3.95), severe −12.65 (3.07), *P* < 0.05)	–	Fibrosis.	GLS, r = 0.421, *P* < 0.001. Multivariate regression (R^2^ 0.35, *P* < 0.05).	–
Ávila-Vanzzini et al. 2016 (21).	Severe AS (*n* = 18).	Patients with more than 50% of PIELV and PIEF had (GLS (mean(SD)): −11.7 (3.3)% vs. -17.1 (1.7)%, *P* < 0.05). Patients with more than 50% fibrosis had significantly lower GLS.	–	Myocardial interstitial fibrosis, Fatty infiltration.	GLS: Fibrosis (R^2^ 0.661, *P* < 0.05).	–
Fabiani et al. 2016 (22).	Severe AS (*n* = 36, Histological analysis; *n* = 23).	GLS % (n = 36) (mean(SD)) −14.0 (3.88).	SSRs (1/s) (mean(SD)) −0.58 (0.17), SSRe (1/s) 0.62 (0.32), SSL (%) − 9.63 (2.97).	Fibrosis, interstitial miRNA-21, plasmatic miRNA-21.	GLS, fibrosis: R^2^ = 0.30 and *P* < 0.05. Interstitial miRNA-21, GLS: R^2^ = 0.34 and *P* < 0.05.	SSL, Fibrosis: R^2^ = 0.36 and *P* < 0.05; SSRs: R^2^ = 0.39 and *P* < 0.001; SSRe: R^2^ = 0.35 and *P* < 0.05. Interstitial miRNA-21, SSL: R^2^ = 0.32 and *P* < 0.05 Plasmatic miRNA-21, SSL: R^2^ = 0.35; *P* < 0.05.
Cameli et al. 2016 (20).	DCM, ICM (*n* = 47).	Patients with extensive fibrosis (> 50%) versus fibrosis(≤50%); GLS, GCS and torsion (mean(SD)) (− 5.4 (2.2) vs − 15.2 (9.1)%, *P* < .0001; − 10.9 (3.1) vs − 16.2 (9.8)%, *P* < 0.05 and 4.2 (1.3) vs 6.6 (2.5)°, respectively).	–	Myocardial fibrosis.	GLS (r = 0.75, *P* < 0.001). GCS and LV torsion (r = 0.61, *P* < 0.05 and r = 0.52, *P* < 0.05, respectively).	–
Escher et al. 2013 (24).	Myocarditis (*n* = 25).	In the acute phase all patients showed a reduction in GLSR (mean(SD)) (0.53 (0.29) 1/s) and GLS (− 8.36 (3.47)%) At follow-up GLS and GLSR were significantly lower in patients with inflammation, in contrast to the patients without inflammation (− 9.4 (1.4) versus − 16.8 (2.0)%, *P* < 0.0001; 0.78 (0.4) versus 1.3 (0.3) 1/s, respectively).	–	Lymphocytic infiltrates, monocytes/macrophages (Mac-1).	GLS; lymphocytic infiltrates (for CD3 *r* = 0.7, *P* < 0.0001, and LFA-1 *r* = 0.8, *P* < 0.0001) but not with monocytes/macrophages (Mac-1).	
Kasner et al. 2013 (25).	Acute myocarditis (*n* = 34).	GLS (mean(SD)) (No myocarditis vs acute myocarditis − 17.86 (3.86) vs − 10.24 (4.12), *P* < 0.05) GLSR (No myocarditis vs acute myocarditis 1.24 (0.26) vs 0.79 (0.27), *P* < 0.05).	–	–	–	–
Mehta et al. 2019 [28].	Cardiac Amyloidosis (*n* = 59)	GLS (mean (SD)) in patients with low-to-moderate amyloid burden versus patients with high amyloid burden − 10.7 (4.9) vs − 6.4 (3.7), *P* < 0.05.	–	–	–	–

*HCM* Hypertrophic cardiomyopathy, *GLS* global longitudinal strain, *SSL* septal longitudinal strain, *GCS* global circumferential strain, *NS* not significant, *AS* aortic stenosis, *PIELV* percentage of infiltrating intra-endocardial lipid vacuoles, *PIEF* percentage of intra-endomyocardial fibrosis, *GLSR* global longitudinal systolic strain rate, *SRe* early systolic strain rate, *SSRs* septal systolic strain rate, *SSRe* septal early diastolic strain rate, *SICAD* small intramural coronary arteriole dysplasia, *HCM*_*MUT*_ sarcomere mutation-positive HCM, *HCM*_*SMN*_ sarcomere mutation-negative HCM, *Mac-1* Macrophage 1 antigen, *LFA-1* Lymphocyte function-associated antigen 1, *MRI* magnetic resonance imaging, *FA-CM* Friedreich ataxia cardiomyopathy

Conversely, pathologies globally affecting the myocardium have been shown to be significantly correlated with deformation parameters. Myocardial fibrosis in patients with advanced heart failure have been linked to the degree of change in LV global longitudinal strain (GLS) [20]. Similarly, correlations have been reported between myocardial fibrosis and GLS in patients with severe aortic stenosis (AS) [21–23]. In patients with myocarditis, GLS and SR parameters of the LV have been shown to be correlated with the degree of cellular infiltrate during the acute phase [24, 25]. However, while global myocardial deformation has proven utility in diagnosis and prognosis [4, 26, 27], segmental deformation may provide tissue characterisation on a more regional level, allowing diagnosis of pathologies with a non-uniform distribution.

There has been only limited investigation into the relationship between deformation and other myocardial pathologies at the tissue level. In cardiac amyloidosis, GLS magnitude is inversely correlated with severity of amyloid deposition on endomyocardial biopsy [28]. Although we could not find direct comparisons with myocardial histology, in Friedreich’s ataxia cardiomyopathy, GLS (but not longitudinal strain rate) was associated with more advanced disease on imaging criteria [29]. To the best of our knowledge, a direct link between the underlying histology and echocardiographic deformation measures has yet to be established in Fabry disease, but impairment of GLS correlates with degree of late gadolinium enhancement on cardiac MRI [30].

### Segmental STE deformation for tissue characterisation

The following sections discussing segmental STE deformation in hypertrophic cardiomyopathy, aortic stenosis, and dilated cardiomyopathy are summarised in Table 2.

**Table 2:** Summary of findings in hypertrophic cardiomyopathy, dilated cardiomyopathy and aortic stenosis

Pathological Condition		Segmental STE Parameter	
		SRS	SRE
Hypertrophic Cardiomyopathy			
	Myocyte hypertrophy	↓^a^	↓
	Myocyte disarray	↓	↓
	Small intramural coronary arteriole dysplasia	↓	↓
	Interstitial fibrosis	↓	↓
Dilated Cardiomyopathy			
	Myocyte diameter	↓	↓↓
	Interstitial fibrosis	NS	NS
Gene expression	Transforming growth factor ß-1	NS	NS
	Collagen type I, Collagen type III	NS	↓
	Titin isoform N2B	NA	↓
	Titin isoforms N2BA	NA	↓ b
	SERCA2a	↓	↓
	Phospholamban	NS	NS
	Phosphorylated Smad2/3	NS	↓
Protein expression	SERCA2a	↓	↓
	Phosphorylated PLB	↓	↓
	Phosphorylated Smad2/3	NS	NS
Aortic Stenosis			
	Interstitial fibrosis	↓	↓
	Tissue miRNA-21 levels	NS	NS

a) Negative correlation between the absolute value of the deformation and the variable; the higher the value of the variable, the lower the deformation.

b) Positive correlation between the absolute value of the deformation and the variable; the higher the value of the variable, the higher the deformation.

Abbreviations: NA, not assessed or data not provided; NS, not statistically significant; SR_S_, systolic strain rate; SR_E_, early diastolic strain rate; STE, speckle tracking echocardiography; SERCA2a, Sarcoplasmic Reticulum Ca^2+^-ATPase

### Hypertrophic cardiomyopathy – histological findings

**Fig. 3:**
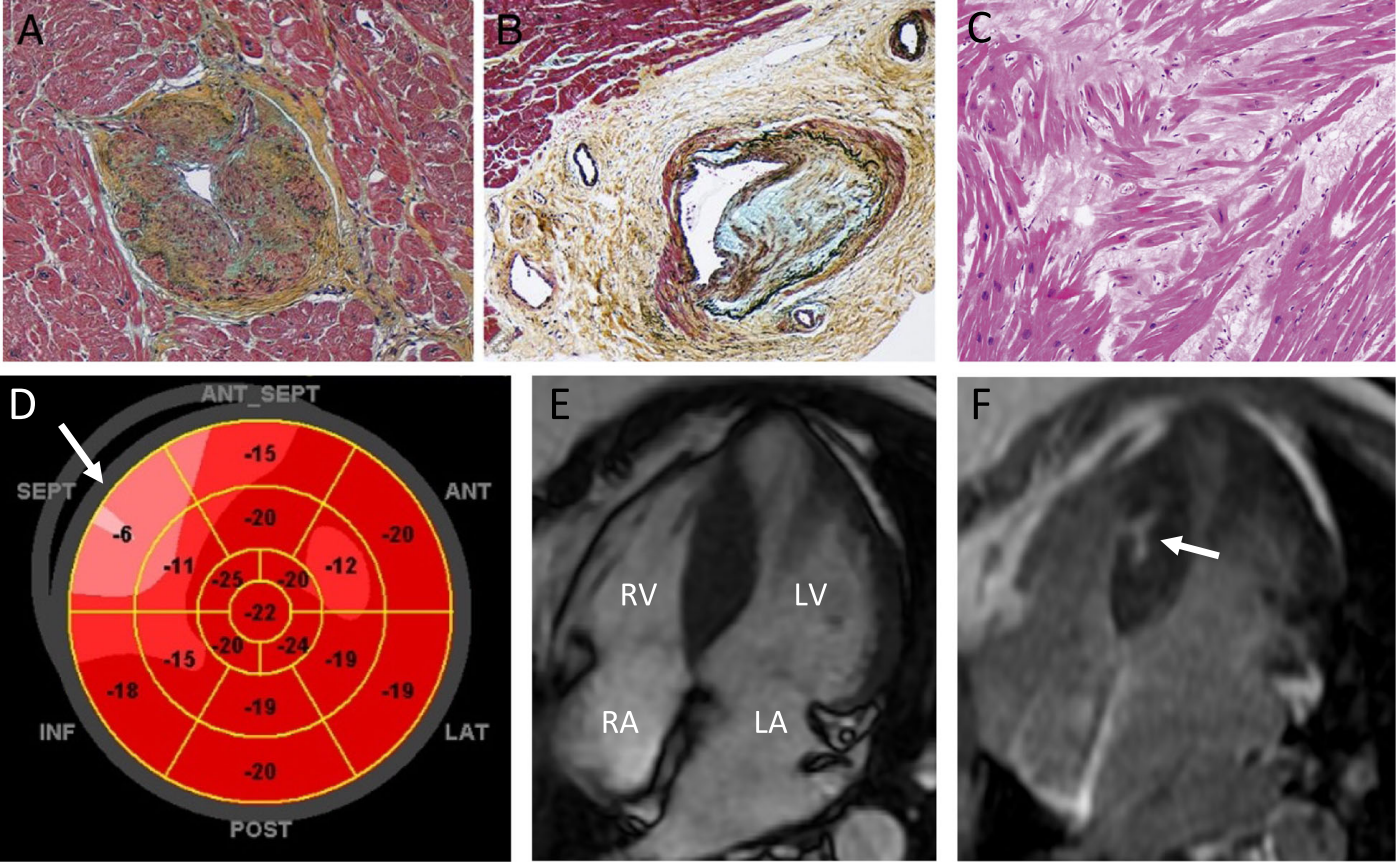
Pathological and imaging findings in hypertrophic cardiomyopathy. The top row shows examples of pathological findings in hypertrophic cardiomyopathy - small intramural coronary arteriole dysplasia, with narrowed lumen (**a** and **b**, reproduced with permission from [54]), and interstitial fibrosis with widespread myocyte disarray (**c**, reproduced with permission from [55]). The bottom row shows imaging findings in a single patient. Speckle tracking echocardiography shows segmental impairment in longitudinal systolic strain, particularly in the base to mid inferior septum (**d**, arrow). Global longitudinal strain was normal, at − 19%. Cardiac MRI shows septal hypertrophy (**e**) with late gadolinium enhancement (**f**, arrow), corresponding to the area of impaired strain. Abbreviations: LA, left atrium; LV, left ventricle; RA, right atrium; RV, right ventricle

HCM is characterised by histopathological changes including hypertrophy of cardiomyocytes, interstitial fibrosis, myocyte disarray, and small vessel disease (small intramural coronary arteriole dysplasia (SICAD)) [31], with examples shown in Fig. 3. Kobayashi et al. [13] investigated the correlation between STE deformation indices and underlying histopathology in 171 patients with HCM undergoing septal myectomy compared with patients with hypertensive heart disease. Comparing the histology of the resected basal septum with SR in the corresponding segment, they found decreasing magnitude of systolic and early diastolic longitudinal SR with increasing myocyte hypertrophy, interstitial fibrosis, SICAD, and fibre disarray. After adjustment for clinical and other echocardiographic variables, a linear relationship between longitudinal systolic SR was seen with myocyte hypertrophy and disarray, but not with SICAD or fibrosis, while early diastolic SR was independently associated with all four conditions.

### Hypertrophic cardiomyopathy – functional findings

The same group correlated STE deformation parameters with in vitro mechanics of isolated trabecular muscle in 122 HCM patients [12], and showed that resting tension (the tension measured when the muscle was mounted on the measurement instrument) and developed tension (the maximum tension developed after lengthening the muscle) were significantly correlated with longitudinal systolic strain and early diastolic SR. In multivariate analysis (which included degree of myocyte disarray and interstitial fibrosis), both systolic strain and early diastolic SR were independently associated with both resting and developed cardiomyocyte tension. Similarly, in a separate study of 46 HCM patients, longitudinal strain in the basal septum was modestly correlated with maximal tension developed by single cardiomyocytes isolated from resected septal myocardium [11].

### Aortic stenosis

STE strain was compared with histological analysis of basal septal myocardial biopsy obtained in 23 patients with AS undergoing aortic valve replacement surgery [22]. Histological assessment of fibrosis included quantitative measurement of fibrosis area and tissue microRNA miR-21 (which has a well-established association with interstitial fibrosis [32]). Myocardial fibrosis percentage area was significantly correlated with the deformation measures of longitudinal systolic strain and SR, and early diastolic SR, in the corresponding segment. Longitudinal systolic strain was also correlated with interstitial miR-21.

### Dilated cardiomyopathy

Samples from hearts explanted from patients with dilated cardiomyopathy (DCM) undergoing heart transplantation provide an invaluable opportunity to analyze STE associations, albeit in a select population. In a very elegant study by Cordero-Reyes et al., histological features, messenger RNA and protein expression, were compared with segmental STE deformation (longitudinal, circumferential and radial strain and SR measured at the LV apex, mid lateral wall, mid septum, and the right ventricular (RV) free wall) in 20 individuals with DCM [14]. A statistically significant association was found between myocyte diameter and longitudinal and circumferential strain and SR – segments with higher myocyte diameters had less deformation. However, in these patients with end-stage DCM, the range of values of fibrosis was minimal, and no association was seen between fibrosis percentage and segmental deformation. Expression of the calcium cycling proteins sarcoplasmic reticulum Ca^2+^ ATPase (SERCA2a) and phospholamban was correlated with segmental deformation measures, but again, likely due to the limited range of values, no association was seen with fibrosis-related mRNA and measured protein expression (collagen type I and III, transforming growth factor ß1, and phosphorylated Smad2/3). The titin isoforms N2B and N2BA tend to relate, respectively, to increased and decreased myocardial stiffness, and the study confirmed this by finding a negative correlation between deformation measures and N2B, and a positive correlation with N2BA. In a separate group of 8 patients, repeated measures were made in patients having LV assist device implantation, and this showed a strong association between change in SERCA2a and titin N2BA, and longitudinal and circumferential strain and SR (but not radial strain).

### Other cardiomyopathies

While segmental STE deformation imaging has an important role in the diagnosis of cardiac amyloidosis [33, 34], only one study assessed the degree of amyloid deposition in left ventricular tissue directly [34]. Segments in hearts from three cardiac transplantation patients was correlated with segmental STE LS; a total of 51 segments from three hearts were assessed, with the higher the degree of deposited amyloid in the cardiac tissue, the worse the segmental longitudinal strain. A single case report comparing an explanted heart in a patient with cardiac sarcoidosis noted a relationship between areas of impaired systolic strain in both ventricles and macro- and microscopic findings of granulomatous disease [35]. Although without histology correlation, in Fabry disease there is similarly a reduction in magnitude of segmental longitudinal strain in areas with disease involvement identified by cardiac MRI [30].

To summarize, the above-mentioned studies comparing STE deformation imaging with tissue analysis in patients with HCM, AS, DCM, and other cardiomyopathies suggest that a combination of LV echocardiographic imaging parameters can be used to better characterise underlying LV tissue architecture and function.

### Right ventricle

In the study by Cordero-Reyes et al. in patients with DCM [14], segmental STE deformation of the RV free wall had similar associations to those seen in the LV, namely increased myocyte diameter was associated with less deformation (longitudinal and circumferential strain as well as systolic and early diastolic SR), and there was no association between deformation measures and interstitial fibrosis or fibrosis related proteins. Increased SERCA2a was associated with more segmental deformation, and, again similar to the LV, there was a negative correlation between deformation measures and titin isoform N2B, and a positive correlation with titin isoform N2BA.

### Left atrium

The left atrium (LA) has three distinct roles in LV filling – in addition to atrial contraction, it acts as a reservoir and a conduit for pulmonary venous return [36]. Atrial cardiomyocytes differ from those in the ventricle in many physiological and functional aspects [36]. These differences, when added to the unique geometry and structure of both atria, make it necessary to study the deformation and mechanics of atria independently from ventricles. Despite having some limitations regarding methodology and reference values [17], STE deformation imaging provides valuable information about atrial mechanics and its correlation with a range of cardiac conditions [36]. A recent consensus document regarding STE deformation analysis was published to standardize the methodology applied for the assessment of these chambers [37]. The difficulty in accurately co-localizing imaging and tissue samples in the atria, as well as the frequent involvement of the entire atrium in a disease process, means that studies tended to focus on global LA deformation.

Her et al. investigated LA deformation in 50 patients with mitral valve disease undergoing mitral valve replacement or repair, 28 of whom had mitral stenosis (MS) [15]. All patients with MS had rheumatic valve disease compared to only four patients with mitral regurgitation (MR), and 75% of patients with MS had atrial fibrillation (AF) compared to 50% of those with MR. LA global strain was significantly correlated with the degree of LA fibrosis, independent of age, rhythm, or type of mitral valve disease. LA strain rate was also univariately associated with fibrosis, but this association was lost on multivariate analysis.

The association between LA STE strain and LA fibrosis was investigated in another study of 46 patients with MR due to mitral valve prolapse undergoing surgery, all of whom were in sinus rhythm [38]. Cameli et al. reported a strong negative correlation between LA global longitudinal systolic strain and LA fibrosis, which remained as an independent association after accounting for LA volume index, LA ejection fraction and LA area. There was a significant correlation between LA global longitudinal systolic strain and endocardial thickness. This was one of the few studies to report standard measures of diagnostic accuracy, and LA longitudinal strain had a higher area under the Receiver Operating Characteristic (ROC) curve than LA volume index or E/e’ ratio as a predictor of LA fibrosis percentage > 50% (area under the ROC curve 0.89).

No studies were found directly correlating segmental or global right atrial deformation with underlying tissue characteristics.

## Discussion

STE deformation imaging can be a powerful imaging modality to investigate many cardiac conditions. However, information on its use as a means to determine local pathological changes remains limited, which is likely related to the scarcity of human tissue samples. Despite this obstacle, the studies discussed in this review establish important links between STE deformation imaging and underlying histopathological changes, which will be valuable for the design of translational cardiac research studies.

Traditional echocardiographic global assessments such as LV volumes and LV EF are excellent for detecting global dilatation and dysfunction. Typically, once overt systolic dysfunction (as detected by impaired LV EF) is present, myocardial deformation analysis offers little additional diagnostic or prognostic information. There remain however many patients where differentiating primary cardiomyopathic processes from, for example, diabetes-related fibrosis or infiltrative processes at an earlier stage of their disease would be clinically important. In selected clinical cases, tissue characterisation is performed with CMR, but this technology is difficult to scale up for routine use. Echocardiography therefore will remain the primary imaging tool for most patients.

### Backscatter and texture analysis

Prior to the advent of CMR, integrated backscatter, which measures the ultrasonic reflectivity of the region of interest, was a major focus of tissue characterisation research [39]. Studies in small series of patients show that backscatter is correlated with fibrosis in undifferentiated cardiomyopathy [40], DCM [41], and AS [42], and correlated with fibrosis, disarray and myocyte diameter in HCM (albeit correlated with RV endomyocardial biopsy findings) [43]. There was also a weak correlation found in a port-mortem study between atrial backscatter measurements and atrial interstitial area [44]. However, backscatter has limited ability to reflect fibrosis in those with lower levels of myocardial fibrosis, such as coronary artery disease [45]. Although there are modest relationships between backscatter and STE deformation measures [46], backscatter has major limitations compared to STE: the need for an intrinsic reference frame, the actual position of the sample volume, the limited validated views, the effect of image setting and the presence of artifacts and other reflectors [17]. However, recent advances in computing power and machine learning may improve reproducibility of intensity-based data [47, 48]. It is likely we will see automated assessment that overcomes some of the challenges associated with current methods.

### Limitations of speckle tracking-echocardiography

STE has a number of well-established limitations: it relies upon good image quality to allow accurate tracking and movement of a region of interest in and out of the image plane will affect strain measurement. This can be overcome using 3D imaging, which has its own limitations. In particular, there can be difficulty optimizing image quality over the whole 3D volume rather than a limited 2D slice, and frame rates are typically lower with 3D imaging. Less experienced sonographers also show lower levels of reproducibility of STE derived strain measures [49]. There also remains intervendor differences in STE derived measures, and this is particularly true of the segmental measures required for regional tissue characterisation [50, 51]. However, initiatives to standardize STE measurement across vendors are likely to provide improved reproducibility [17, 50, 51]. Finally, STE deformation only provides indirect assessment of tissue features as it relies on a change in tissue function. While this has the potential to be specific for certain conditions [52], in general, it will only allow detection of tissue abnormalities without specification of the cause of these– for example, impaired strain is unlikely to be able to distinguish amyloid infiltration from fibrosis on a segmental level.

## Conclusions

Beyond global assessment of myocardial and atrial function, segmental STE deformation measures have limited, but definite, evidence of providing information on the underlying tissue. These measures should be considered for use as surrogate outcomes in early stage clinical trials, with the aim of translating basic research findings to a stage where they can be tested in clinical outcome-based trials.

To move echocardiographic tissue characterisation into clinical utility, we believe the following four steps must be made. First, given the relatively small body of literature available, further research with larger numbers of participants and more in-depth tissue assessment (rather than just a focus on fibrosis alone) is required to provide the robust evidence base needed to rely on these surrogate measures. In particular, there is a distinct lack of studies focusing on the atria, which is especially important in the current context of increasing numbers of patients with atrial fibrillation. Second, formal measures of diagnostic accuracy should be reported, rather than simple correlations. Third, a method of integrating information including global function (such as the complementary information provided by LV shape, volume, EF and GLS) and segmental measures should be developed to allow more advanced phenotyping [53]. As Captur et al. have pointed out [2], based on preclinical studies there should be thousands of cardiomyopathies rather the limited number we diagnose now. Finally, it is possible that STE deformation will fail to provide the level of detail required. Now may be the time to redirect research efforts toward new methods of echocardiographic tissue characterisation, filling the void left after the decline of backscatter research.

## Data Availability

Not applicable.

## Abbreviations

AF: Atrial Fibrillation
AS: Aortic Stenosis
CMR: Cardiac Magnetic Resonance Imaging
DCM: Dilated Cardiomyopathy
GLS: Global Longitudinal Strain
HCM: Hypertrophic Cardiomyopathy
LA: Left Atrium
LV: Left Ventricle
miRNA: MicroRNA
MR: Mitral Regurgitation
mRNA: Messenger Ribonucleic Acid
MS: Mitral Stenosis
PLB: Phospholamban
RA: Right Atrium
ROC: Receiver Operating Characteristic
RV: Right Ventricle
SERCA2a: Sarcoplasmic Reticulum Ca2 + −ATPase
SICAD: Small Intramural Coronary Arteriole Dysplasia
SR: Strain Rate
STE: Speckle Tracking Echocardiography
TGFß1: Transforming Growth Factor beta 1
